# Characterization of chondroitin sulfate in stem cells derived from umbilical cord blood in rats

**DOI:** 10.1371/journal.pone.0262854

**Published:** 2022-01-25

**Authors:** Keiko Nakanishi, Kyohei Higashi, Toshihiko Toida, Masato Asai

**Affiliations:** 1 Department of Disease Model, Institute for Developmental Research, Aichi Developmental Disability Center, Kasugai, Aichi, Japan; 2 Department of Pediatrics, Central Hospital, Aichi Developmental Disability Center, Kasugai, Aichi, Japan; 3 Faculty of Pharmaceutical Sciences, Tokyo University of Science, Noda, Chiba, Japan; 4 Center for Preventive Medical Sciences, Chiba University, Chuo-ku, Chiba, Japan; Universidade Federal do Rio de Janeiro, Instituto de Bioquimica Medica, BRAZIL

## Abstract

Chondroitin sulfate (CS) and its isomeric variant, dermatan sulfate (DS), are complex glycosaminoglycans (GAGs) which are ubiquitous components of the extracellular matrix in various tissues including the brain. CS and/or DS are known to bind to a variety of growth factors and regulate many cellular events such as proliferation and differentiation. Although the biological activities of CS and/or DS towards neural stem/progenitor cells (NSPCs) have been well investigated, the CS and/or DS of hematopoietic stem cells (HSCs) have not been fully characterized. Here, we analyzed GAGs on mononuclear cells of rat umbilical cord blood cells (UCB-MNCs). CS was detected in vascular intima and media of rat umbilical cord at embryonic day 19 (E19) by immunohistochemistry. The stem-cell-enriched-UCBCs (SCE-UCBCs), which were expanded from rat UCB-MNCs, expressed CS. CS chains are composed of repeating disaccharide units, which are classified into several types such as O-, A-, B-, C-, D-, and E-unit according to the number and positions of sulfation. A disaccharide composition analysis revealed that CS and/or DS were abundant in rat UCB-MNCs as well as in their expanded SCE-UCBCs, while the amount of heparan sulfate (HS) was less. The degree of sulfation of CS/DS was relatively low and the major component in UCB-MNCs and SCE-UCBCs was the A-unit. A colony-forming cell assay revealed that the percentage of colony-forming cells decreased in culture with CS degradation enzyme. The CS and/or DS of UCBCs may be involved in biological activities such as stem cell proliferation and/or differentiation.

## Introduction

Chondroitin sulfate (CS) and/or dermatan sulfate (DS) are the major constituents of the extracellular matrix of the central nervous system (CNS) as well as other tissues, and are involved in various cellular events in the formation and maintenance of the neural network in the CNS [1–4]. The CS chain consists of repeating disaccharide units of glucuronic acid (GlcA) and *N*-acetylgalactosamine (GalNAc), which in mammals are commonly sulfated on GalNAc residues, and is highly heterogeneous in structure [5]. There are several CS-disaccharide units with different numbers and positions of sulfation; namely, O, A, B, C, D, and E (S1 Fig in S1 File), although the GlcA-containing B-unit has been reported only in shark skin [6]. Of these disaccharide units, D and E contain two sulfate residues, and CS polysaccharides rich in these highly sulfated disaccharides (CS-D and -E, respectively) have been shown to bind to several growth factors [7, 8] and to be involved in neurite outgrowth [9, 10], neural stem cell proliferation [3], and neuroprotection [11]. Dermatan sulfate (DS, formerly CS-B), another isomeric variant of CS, has iduronic acid (IdoA)-containing disaccharide units instead of GlcA [4, 6]. DS is also known to promote proliferation [3] and differentiation of neural stem cells via interaction with a wide range of growth factors and neurotrophic factors [4]. There are six major DS-disaccharide units which are characterized by their positions of sulfation, namely, iO, iA, iB, iC, iD, and iE [4]. Endogenous CS and DS in mammalian tissues are highly complex and heterogenous in their structure and sulfation pattern, and are often observed in CS/DS hybrid chains [6].

A novel approach, such as use of stem cell therapy, has been anticipated for diseases for which there are no effective cures [12]. Among several stem cell sources, umbilical cord blood cells (UCBCs) offer one of the most suitable materials, as they can be readily obtained at birth and can be administered intravenously. There is increasing evidence that human UCBCs have a favorable effect in treating hypoxia-ischemia (HI) brain injury in neonatal rat/mouse models [13–16]. We have also shown that intraperitoneal administration of stem-cell-enriched umbilical cord blood cells (SCE-UCBCs) expanded from rat UCBCs attenuated HI brain injury in neonatal rats [17]. Wharton’s jelly, a gelatinous substance within the umbilical cord, is composed of extracellular matrix such as hyaluronic acid and CS [18]. CS and/or DS could conceivably be present in UCBCs. Although the biological activities of CS/DS towards neural stem/progenitor cells (NSPCs) have been well investigated [3, 4, 6], the CS/DS composition and/or characteristics in UCBCs and hematopoietic stem cells (HSCs) have not been fully investigated.

In the present study, we examined the existence of CS in UCBCs and analyzed the disaccharide composition of CS/DS and heparan sulfate (HS). Results showed that the degree of sulfation of CS/DS was relatively low and that the major component was the A-unit in MNCs from UCBCs.

## Materials and methods

All experimental animal protocols in the present study were approved by the Review Board of the Institute for Developmental Research, Aichi Developmental Disability Center, and were carried out according to the guidelines for animal research of the Neuroscience Society of Japan to minimize the number of animals used as well as their suffering.

### Immunohistochemical procedures

Immunohistochemical procedures were performed as described previously [19]. Pregnant rats were deeply anesthetized using isoflurane inhalation and the placentas of the pups on embryonic day 19 (E19) were excised and fixed with 4% paraformaldehyde in 0.1 M phosphate buffer. Placenta were dehydrated, embedded in paraffin, and cut into 5-μm-thick serial sections. After deparaffinization, antigen retrieval was performed by heating the sections at more than 90°C for 20 min in 10 mM citrate buffer (pH 6.0). After digestion with or without protease-free Chase ABC (1 U/mL, Seikagaku Corporation) at 37°C for 2 h, sections were incubated in blocking solution, and in solution with primary antibodies (anti-CS (CS-56), mouse IgM, Seikagaku Corporation; mouse monoclonal anti-CS-A (2H6), Seikagaku Corporation). After endogenous peroxidase activity was inhibited, the sections were then treated with anti-mouse IgM-conjugated peroxidase (Kirkegaard & Perry Laboratories, Inc.), followed by peroxidase detection for 10 min (0.01% 3,3′-diaminobenzidine and 0.01% H_2_O_2_).

### Isolation and culture of rat mononuclear cells (MNCs) derived from UCBCs

MNCs were obtained from UCBCs of rat fetuses at E19 as described previously [17]. MNCs were isolated, expanded, and then cryopreserved. Details of the isolation and expansion are described in the S1 File. When the cell number had increased 16 times, about 20% of cells were found to be colony-forming cells as determined by a colony-forming unit-granulocyte, macrophage (CFU-GM) assay after 10 days in culture, although only 0.9% of cells in freshly isolated MNCs of UCBCs were colony-forming [17]. Hereafter, we refer to the expanded cells as stem-cell-enriched UCBCs (SCE-UCBCs).

MNCs of peripheral blood were obtained from 8-week-old adult male rats. We used male rats to avoid effects of the menstrual cycle and/or sex hormones. After the rats were deeply anesthetized using isoflurane inhalation, blood was obtained from the cardiac chamber (usually 7–10 mL/ individual). MNCs were isolated similar to the method used for MNCs of UCBCs as described above. The isolated cells (usually 0.8–1.7 x 10^7^ cells/individual) were then cryopreserved in Cellbanker-3 (Nippon Zenyaku Kogyo Co., Ltd.) until use.

### Immunocytochemical procedures

Immunocytochemical procedures were performed as described previously [17]. Briefly, cells were collected by pipetting with PBS and plated on coverslips coated with poly-L-lysine (Sigma-Aldrich) and fixed. After incubation in the digestion buffer with or without protease-free Chase ABC (1 U/mL, Seikagaku Corporation) at 37°C for 2 h, cells were incubated in blocking solution containing 2% bovine serum albumin, 2% horse serum, and 2% goat serum, and in primary antibody (unsulfated CS stub antibody, 1B5, mouse IgG1, Seikagaku Corporation) at 4°C overnight. The 1B5 antibody recognizes a disaccharide neoepitope generated at the non-reducing terminal of CS chains that were pre-digested with Chase ABC [20]. After three washes with Tris-buffered saline, the cells were then treated with Alexa Fluor 488-conjugated anti-mouse IgG1 (Molecular Probes). The nuclei of cells were counterstained with 4’,6’-diamidino-2-phenylindole (DAPI, Sigma).

For CD133 antibody, after incubation in blocking solution containing 10% donkey serum at room temperature for 1h, cells were incubated in primary antibody (CD133, rabbit, Abcam) at 4°C overnight. After three washes, the cells were then treated with Cy3-conjugated anti-rabbit IgG (Jackson ImmunoResearch) and DAPI (Sigma).

Quantification of immunocytochemical staining was described in S1 File.

### Quantitative analyses of GAGs from MNCs of UCBCs and SCE-UCBCs, and from MNCs of adult blood

A disaccharide composition analysis of GAGs was performed as described previously [21]. Briefly, thawed cells (0.4–2.2 x 10^7^ cells/ preparation, S1 Table in S1 File) were homogenized with 4 volumes of acetone overnight, and the precipitates obtained by centrifugation were proteolyzed at 45°C with actinase E (10 mg/g dry powder) in 50 mM Tris acetate (pH 8.0) for 18 h. Microscale isolation of GAGs was performed according to the method of Zhang et al. [22]. Details of isolation were described in S1 File. Protein contents were determined by the method of Lowry et al. [23].

We used sufficient amounts of enzymes (chondroitinases and heparinases) that were capable of cleaving more than 2 μg of standard material. As shown in S1 and S2 Tables in S1 File, the CS/DS and HS of each preparation was 10.9–950 and 0–287 ng/sample, respectively. Since each sample contained less than 1 μg of CS/DS or HS, all chains of CS/DS or HS in the present study would be expected to be completely digested.

### CFU-GM assay

A CFU-GM assay was performed as described previously [17]. A colony-forming cell assay for rat cells (MethoCult GFR3774, StemCell Technology) is recommended for the detection and quantification of rat CFU-GM progenitors in bone marrow samples. Briefly, 0.3 mL of cell suspension (2,500 cells/mL for SCE-UCBCs, 1 x 10^5^ cells/mL for UCB-MNCs, and 1 x 10^6^ cells/mL for adult MNCs) was added to 3 mL of MethoCult. Using a 2.5 mL syringe, 1.1 mL of the MethoCult mixture was dispensed into each of two wells of a 6-well plate and cultured. After 10 days, the cultures were photographed using a stereo microscope (M165FC, Leica), and the number of colonies in each of the two wells was counted using a Photoshop counting tool (Adobe) and then averaged. The percentage of colony-forming cells was calculated as the number of colonies divided by the number of seeded cells. The experiments were repeated 3 times (n = 3).

UCB-MNCs were cultured with PBS, Chase ABC (0.2 U/mL, Seikagaku Corporation), CS-A (from whale cartilage, 80% of which consists of ΔDi-4S (A-unit), 100 μg/mL, Seikagaku Corporation), or CS-E (from squid cartilage, 60–65% of which consists of ΔDi-di4, 6S (E-unit), 100 μg/mL, Seikagaku Corporation) (Fig 5). Data are shown as the percentage of the control (PBS). The experiments were repeated 4 times and statistically analyzed.

### Statistical analysis

All data are presented as means ± standard error (SEM). Statistical differences were compared using unpaired t test or ANOVA followed by Tukey-Kramer multiple comparison test. A p<0.05 was considered statistically significant. All analyses were performed using Instat 3 (Graph Pad Corp.).

## Results

### Localization of CS to the vasculature of the umbilical cord

**Fig 1:**
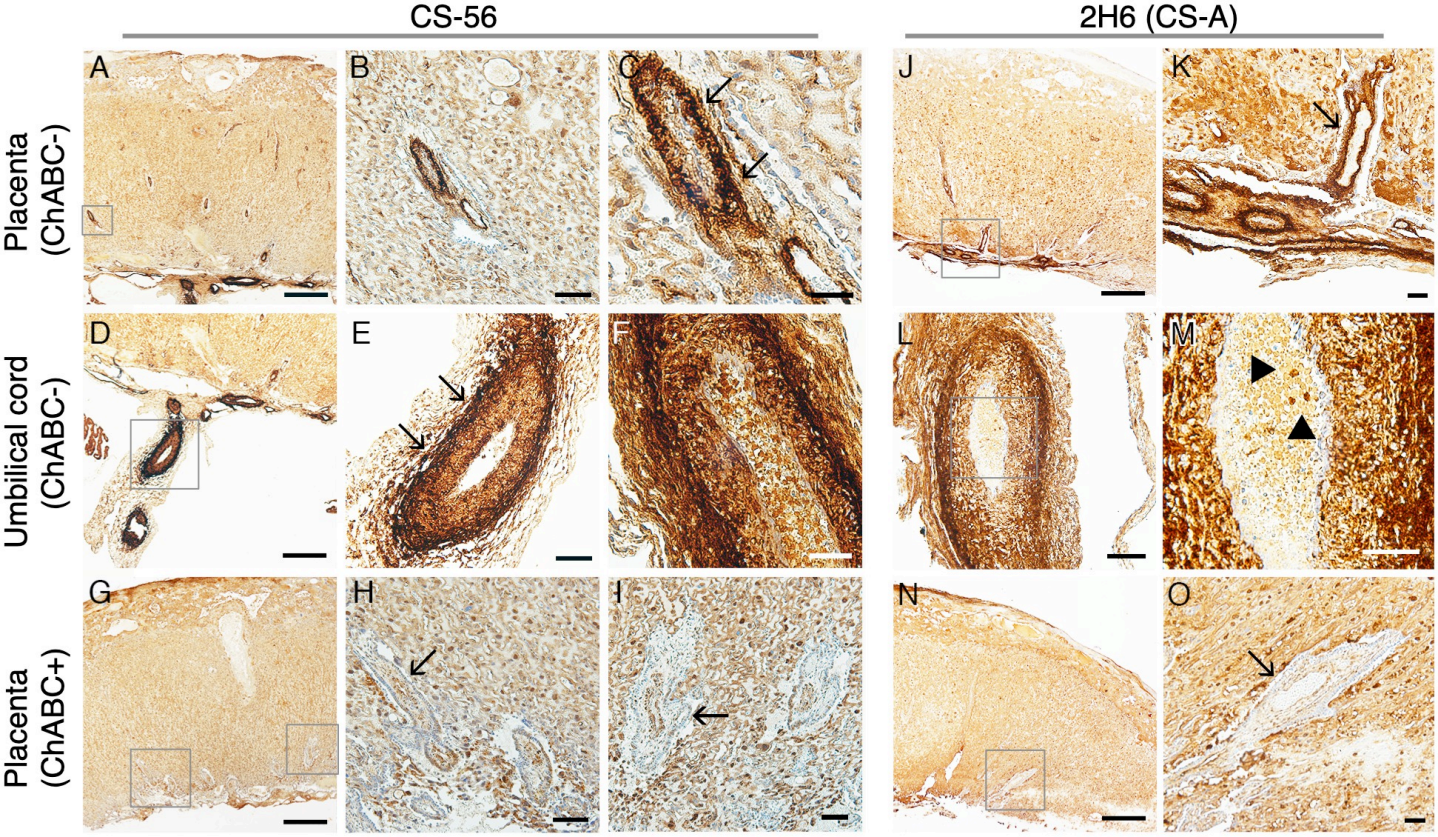
Immunohistochemical staining of chondroitin sulfate in rat placenta and umbilical cord. (A-I) Representative photomicrographs of the staining of anti-CS-56 antibody in rat placenta (A-C) and umbilical cord (D-F). Boxes A, D, and G are presented as the photographs B, E, and H/I, respectively. Fig 1C is a higher magnification image of Fig 1B. Immunoreactivity of CS was observed, especially in intima and media of vasculature (A-F, arrows in C and E), which disappeared by CS removal on treatment with Chase ABC (G-I, arrow in H and I). (J-O) Representative photomicrographs of the staining of anti-CS-A (2H6) antibody in rat placenta (J, K) and umbilical cord (L, M). Boxes in J, L, and N are presented as photographs of K, M, and O, respectively. Similarly, immunoreactivity was seen in intima and media of vasculature (J-M, arrow in K), and it disappeared by CS removal with Chase ABC (N, O, arrow in O). Bar, 500 μm (A, D, G, J, N), 100 μm (B, E, H, L), 50 μm (C, F, I, K, M, O).

In order to identify the histological distribution of CS, we first performed immunohistochemistry of placenta and umbilical cord of embryonic rats at E19. Immunoreactivity with the monoclonal antibody anti-CS (CS-56) was observed in intima and media of vasculature in the umbilical cord (Fig 1D–1F, arrows in Fig 1E) and in their conceivable branches in the placenta (Fig 1A–1C, arrows in Fig 1C), and this reactivity disappeared by removing CS on treatment with Chase ABC (ChABC), a CS/DS digestion enzyme (Fig 1G–1I, arrow in Fig 1H and 1I). Similar results were obtained using another monoclonal antibody, anti-CS-A (2H6, Seikagaku Corporation, Fig 1J–1O). Moreover, Fig 1M shows the immunoreactivity of CS in a small subpopulation of UCBCs within the vasculature of the umbilical cord (arrowheads in Fig 1M). The 2H6 immunoreactivity also disappeared by removing CS with Chase ABC (ChABC, Fig 1N and 1O, arrow in Fig 1O).

### Presence of CS in expanded stem-cell-enriched-UCBCs (SCE-UCBCs)

We isolated the MNCs of UCBCs from E19 rat embryos and expanded these cells using several growth factors as described (S1 File) [17]. We refer to the expanded cells as stem-cell-enriched UCBCs (SCE-UCBCs) [17].

**Fig 2:**
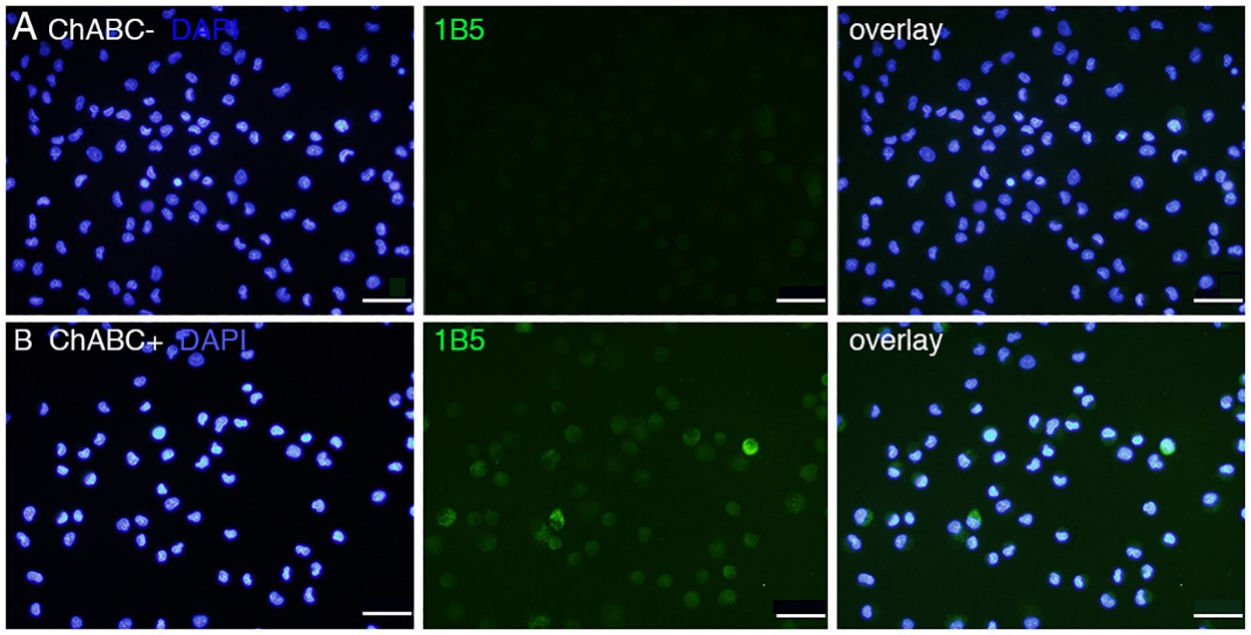
Immunocytochemical staining of CS stub in expanded SCE-UCBCs. Representative photomicrographs of the staining of 1B5 (unsulfated CS stub) monoclonal antibody in expanded SCE-UCBCs. Immunoreactivity was only observed in the cells pre-digested with Chase ABC (B), and not in the cells not digested with Chase ABC (A, see Methods). blue, DAPI; green, 1B5 antibody. Bar, 50 μm.

We performed immunocytochemistry using a CS stub antibody (1B5, mouse IgG1, Seikagaku Corporation). The 1B5 antibody recognizes unsulfated unsaturated disaccharide neoepitopes generated at the non-reducing terminal of CS chains that have been pre-digested with Chase ABC [20]. As shown in Fig 2, a small number of 1B5-positive cells was detected in SCE-UCBCs only after treatment with Chase ABC (Fig 2B) and not in SCE-UCBCs without Chase ABC treatment (Fig 2A). The percentage of 1B5-positive cells was 6.5±1.9% (n = 4). This result indicates that CS chains including the O-unit are present in some SCE-UCBCs. We tried immunostaining using the 2B6 antibody (the stub antibody against A-unit), but were unsuccessful with the cultured cells even after pre-digestion with Chase ABC.

### Disaccharide composition of CS/DS in GAG derived from SCE-UCBCs and MNCs

CS and DS polysaccharides are generally present as side chains of proteoglycans. CS/DS can be digested by Chase enzymes in an eliminative fashion yielding a disaccharide unit with an unsaturated bond at the nonreducing end [24]. To confirm the presence of CS/DS in UCBCs, we analyzed the disaccharide composition of CS/DS in SCE-UCBCs using HPLC.

**Fig 3:**
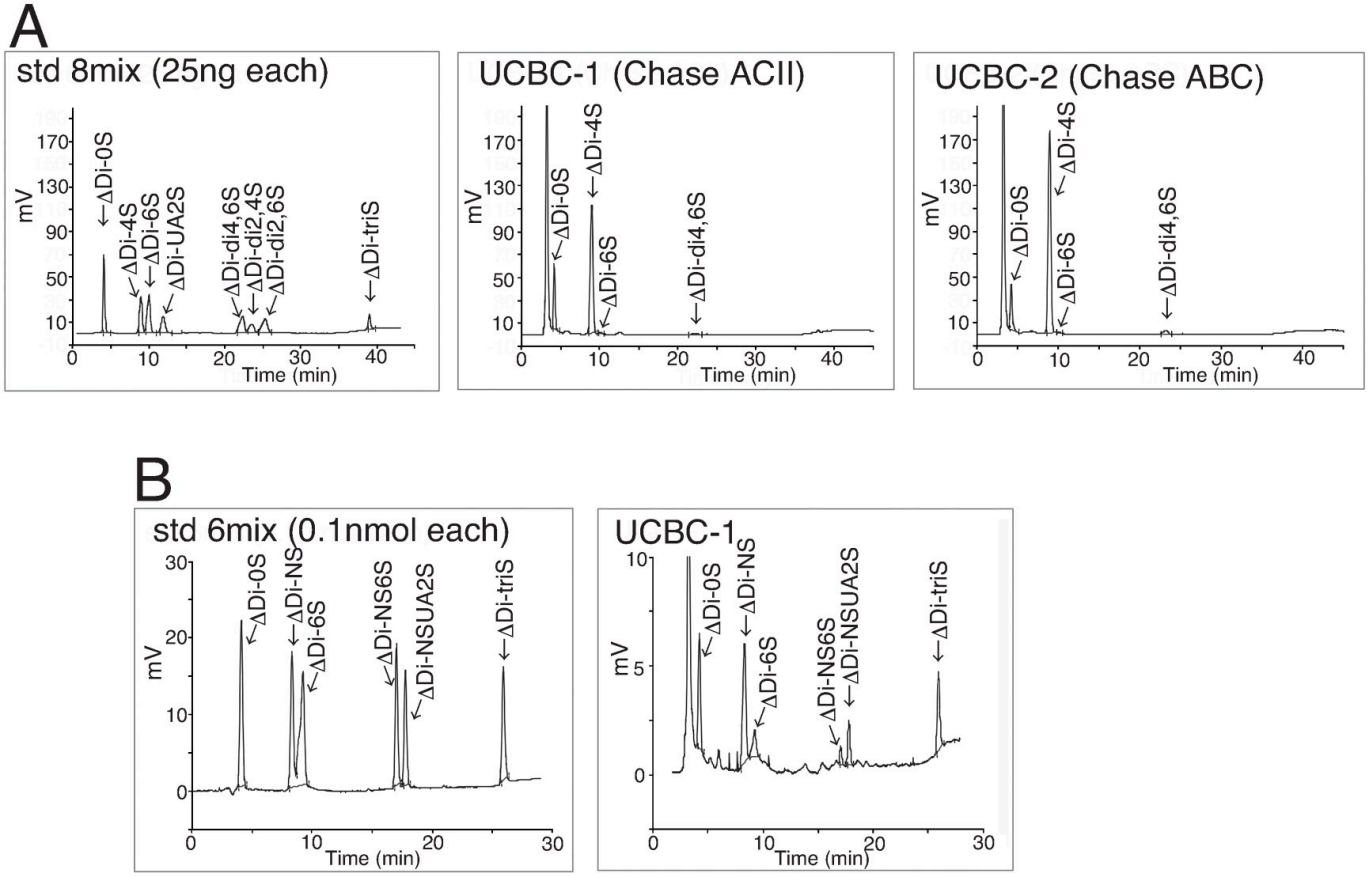
CS/ HS disaccharide composition analysis by HPLC. (A) CS disaccharide composition analysis. Representative chromatogram of the standard (left panel), the digestion of SCE-UCBCs with Chase ACII (UCBC-1, middle panel), and that with Chase ABC (UCBC-2, right panel). ΔDi-0S, deoxy-α-L-*threo*-hex-4-enopyranosyluronic acid (ΔUA) (1→3) *N*-acetylgalactosamine (GalNAc); ΔDi-4S, ΔUA(1→3)GalNAc4S, where S is sulfo; ΔDi-6S, ΔUA (1→3) GalNAc6S; ΔDi-UA2S, ΔUA2S (1→3) GalNAc; ΔDi-di4,6S, ΔUA (1→3) GalNAc4S6S; ΔDi-di2,4S, ΔUA2S (1→3) GalNAc4S; ΔDi-di2,6S, ΔUA2S (1→3) GalNAc6S; ΔDi-triS, ΔUA2S (1→3) GalNAc4S6S. (B) HS disaccharide composition analysis. Representative chromatogram of the standard (left panel) and digestion of SCE-UCBCs with heparinases (UCBC-1, right panel). ΔDi-0S, ΔUA (1→4) *N*-acetylglucosamine (GlcNAc); ΔDi-NS, ΔUA (1→4) *N*-sulfated glucosamine (GlcNS); ΔDi-6S, ΔUA (1→4) GlcNAc6S; ΔDi-NS6S, ΔUA (1→4) GlcNS6S; ΔDi-NSUA2S, ΔUA2S (1→4) GlcNS; ΔDi-TriS, ΔUA2S (1→4) GlcNS6S.

At first, CS/DS purified from 1.5 x 10^7^ cells of SCE-UCBCs was treated with Chase ACII or Chase ABC, and then subjected to HPLC. Fig 3A shows representative chromatograms of the standard (std, left panel), an SCE-UCBC sample digested with Chase ACII (UCBC-1, middle panel), and an SCE-UCBC sample digested with Chase ABC (UCBC-2, right panel). DS can be digested into disaccharides by Chase ABC but not by Chase ACII [25]. Since the peak that eluted at around 22 min is seen in the chromatogram when the sample was digested with Chase ACII (middle panel) as well as Chase ABC (right panel), this peak is considered to be ΔDi-di4, 6S (E-unit) rather than DS (ΔDi-di2,4S, iB-unit, formerly B-unit). The peaks of the highly sulfated disaccharide units (ΔDi-di2,4S, ΔDi-di2,6S, and ΔDi-triS) except for ΔDi-di4,6S, were not observed in UCBC samples (middle and right panel). Table 1 shows the disaccharide composition of CS/DS in SCE-UCBCs digested with Chase ACII or Chase ABC. The amount of the A-unit on digestion with Chase ABC (which digests CS and DS) was larger than that on digestion with Chase ACII (which digests only CS)(Chase ABC, 84.3% of 408.2 ng/mg, which is 344.1 ng/mg protein vs. Chase ACII, 75.7% of 330.4 ng/mg, which is 250.1 ng/mg protein), indicating the presence of some iduronic acid (IdoA)-containing disaccharide (iA-unit, one of the DS disaccharides) in the SCE-UCBCs. In other words, SCE-UCBCs presumably harbor CS/DS hybrid chains.

**Table 1:** Disaccharide composition of CS/DS in GAG from SCE-UCBCs digested with Chase ACII or Chase ABC.

Chase	Sample	Unsaturated disaccharide units								ng/mg protein
		ΔDi-0S (%)	ΔDi-4S (%)	ΔDi-6S (%)	ΔDi-UA2S (%)	ΔDi-di4,6S (%)	ΔDi-di2,4S (%)	ΔDi-di2,6S (%)	ΔDi-triS (%)	
ACII	UCBC1	21.7	75.7	0.5	ND	1.9	ND	ND	ND	330.4
ABC	UCBC2	12.7	84.3	0.3	ND	2.7	ND	ND	ND	408.2

ΔDi-0S, deoxy-α-L-*threo*-hex-4-enopyranosyluronic acid (ΔUA) (1→3) *N*-acetylgalactosamine (GalNAc); ΔDi-4S, ΔUA(1→3)GalNAc4S, where S is sulfo; ΔDi-6S, ΔUA (1→3) GalNAc6S; ΔDi-UA2S, ΔUA2S (1→3) GalNAc; ΔDi-di4,6S, ΔUA (1→3) GalNAc4S6S; ΔDi-di2,4S, ΔUA2S (1→3) GalNAc4S; ΔDi-di2,6S, ΔUA2S (1→3) GalNAc6S; ΔDi-TriS, ΔUA2S (1→3) GalNAc4S6S; ND, not detected.

Next, GAGs from expanded SCE-UCBCs, freshly isolated MNCs from rat UCBCs (UCB-MNC), and freshly isolated MNCs from peripheral blood of adult rats (Adult-MNC) were digested with Chase ABC and their CS/DS disaccharide compositions were analyzed by HPLC (Table 2). Since the number of MNCs from rat UCBCs was usually less than 1 x 10^6^ cells per litter [17], UCBC-MNCs were collected from 6 litters and their combined GAGs were analyzed. The degree of sulfation of CS/DS was relatively low and the major component was the A-unit (ΔDi-4S, more than 80%) in three preparations. A small percentage of the O-unit (ΔDi-0S, 4–13%) was also detected. The highly sulfated E-unit (ΔDi-di4,6S) was barely detected in SCE-UCBCs (3.3±0.4%, Table 2). The C-unit (ΔDi-6S) was detected in adult MNCs (9.0±4.4%) and not in UCB-MNCs (Table 2).

**Table 2:** Disaccharide composition of CS/DS in GAG from UCBCs and MNCs.

Sample	Unsaturated disaccharide units								ng/mg protein
	ΔDi-0S (%)	ΔDi-4S (%)	ΔDi-6S (%)	ΔDi-UA2S (%)	ΔDi-di4,6S (%)	ΔDi-di2,4S (%)	ΔDi-di2,6S (%)	ΔDi-triS (%)	
SCE- UCBC^a^	13.2±0.6	83.1±0.7	0.4±0.1	ND	3.3±0.4	ND	ND	ND	544±91
UCB- MNC^b^	13.7	86.3	ND	ND	ND	ND	ND	ND	238
Adult MNC^c^	4.4±1.9	86.7±2.5	9.0±4.4	ND	ND	ND	ND	ND	154±40

^a^stem-cell-enriched umbilical cord blood cells (n = 3),

^b^mononuclear cells derived from rat umbilical cord blood (collected from 70 pups),

^c^mononuclear cells derived from peripheral blood of adult rat (n = 3).

ΔDi-0S, deoxy-α-L-*threo*-hex-4-enopyranosyluronic acid(ΔUA)(1→3)*N*-acetylgalactosamine (GalNAc); ΔDi-4S, ΔUA(1→3)GalNAc4S, where S is sulfo; ΔDi-6S, ΔUA (1→3) GalNAc6S; ΔDi-UA2S, ΔUA2S (1→3) GalNAc; ΔDi-di4,6S, ΔUA (1→3) GalNAc4S6S; ΔDi-di2,4S, ΔUA2S (1→3) GalNAc4S; ΔDi-di2,6S, ΔUA2S (1→3) GalNAc6S; ΔDi-TriS, ΔUA2S (1→3) GalNAc4S6S; ND, not detected.

The HS content of GAGs derived from SCE-UCBCs and MNCs was much smaller than the CS content (Fig 3B, Table 3). There was a significant difference between the CS and HS content in SCE-UCBCs (544±91 vs. 147±20 ng/mg protein, respectively, n = 3, p<0.05, Tables 2 and 3, S1 and S2 Tables in S1 File). We did not detect any HS in MNCs of UCBCs (Table 3).

**Table 3:** Disaccharide composition of HS in GAG from UCBCs and MNCs.

Sample	Unsaturated disaccharide units						ng/mg protein
	ΔDi-0S (%)	ΔDi-NS (%)	ΔDi-6S (%)	ΔDi-NS6S (%)	ΔDi-NSUA2S (%)	ΔDi-triS (%)	
SCE- UCBC^a^	21.2±2.4	35.3±4.6	5.9±0.6	3.5±0.8	13.3±2.6	20.8±3.3	147±20
UCB- MNC^b^	ND	ND	ND	ND	ND	ND	ND
Adult MNC^c^	53.9±20.8	46.1±20.8	ND	ND	ND	ND	7.5±0.9

^a^stem-cell-enriched umbilical cord blood cells (n = 3),

^b^mononuclear cells derived from rat umbilical cord blood(collected from 70 pups),

^c^mononuclear cells derived from peripheral blood of adult rat (n = 3).

ΔDi-0S, ΔUA (1→4) *N*-acetylglucosamine (GlcNAc); ΔDi-NS, ΔUA (1→4) *N*-sulfated glucosamine (GlcNS); ΔDi-6S, ΔUA (1→4) GlcNAc6S; ΔDi-NS6S, ΔUA (1→4) GlcNS6S; ΔDi-NSUA2S, ΔUA2S (1→4) GlcNS; ΔDi-TriS,ΔUA2S (1→4) GlcNS6S; ND, not detected.

**Fig 4:**
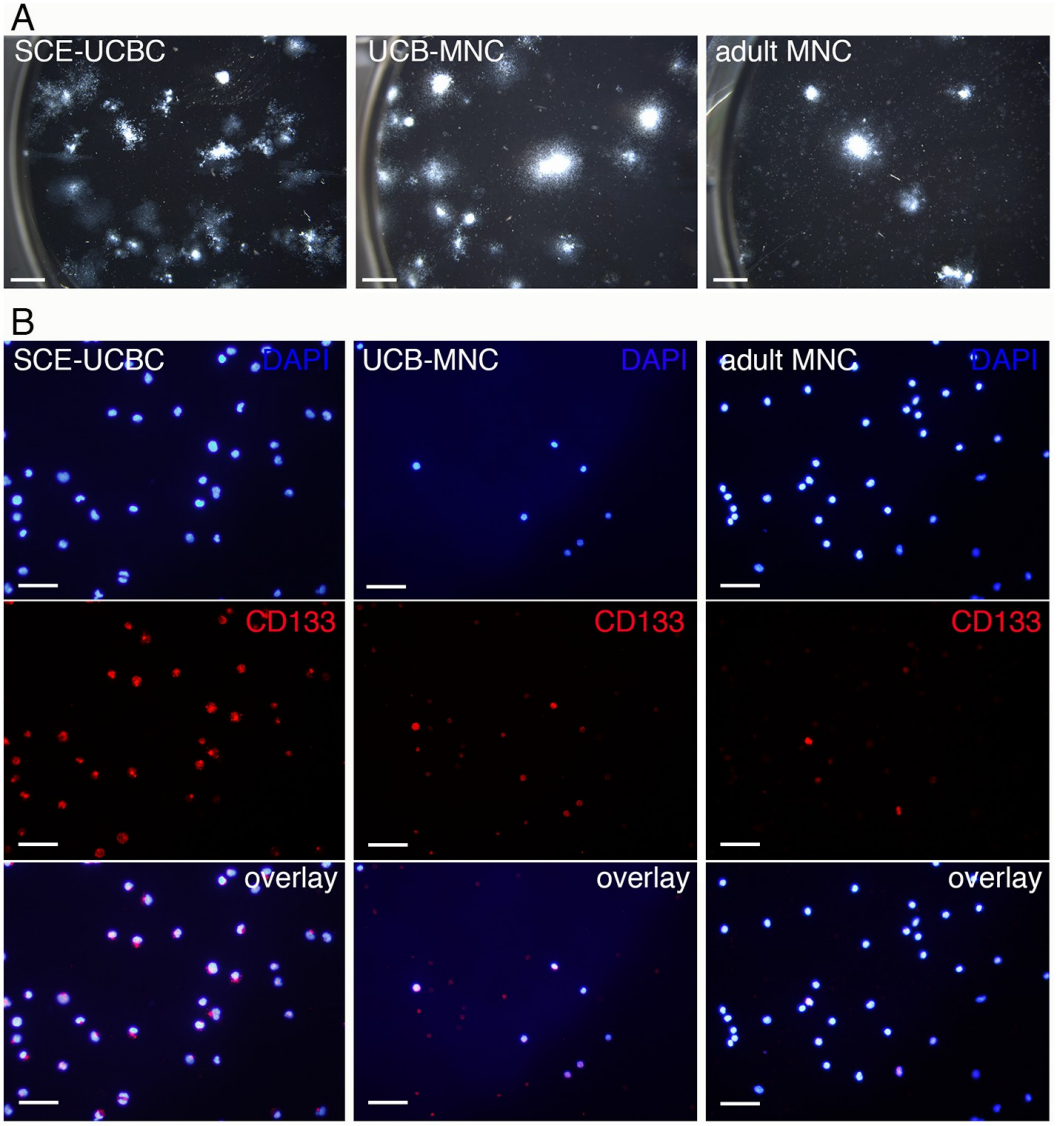
Colony-forming cell assay and CD133 immunostaining of SCE-UCBC, UCB-MNC, and adult MNC. (A) Representative photographs of a CFU-GM assay of SCE-UCBCs, UCB-MNCs, and adult MNCs. Note that the number of seeded cells was different (250 cells/well for SCE-UCBCs, 1x 10^4^ cells/well for UCB-MNCs, and 1 x 10^5^ cells/well for adult MNCs, see Methods). Bar, 2 mm. (B) Representative photomicrographs of immunostaining against CD133 antibody. Upper panel; DAPI, middle panel, CD133, lower panel, overlay. Bar, 50 μm.

In our previous study, about 20% of the total number of SCE-UCBCs were colony-forming cells, whereas the percentage of colony-forming cells in MNCs from UCBCs was less than 1% (Table 4) [17]. We performed a CFU-GM assay using adult MNCs (Fig 4A) and found that the percentage of colony-forming cells in adult MNCs was 0.035±0.0075% (n = 3, Table 4). Consequently, the expected number of colony-forming cells in each preparation was hugely different (296, 3.42, and 0.36 x 10^4^ cells in SCE-UCBCs, UCB-MNCs, and adult MNCs, respectively, Table 4). In addition, we investigated another characterization of cells using CD133 antibody (stem cell marker, Fig 4B). We previously found that the percentages of CD133^+^ cells in UCB-MNCs and SCE-UCBCs were 54.7±10.8% and 77.0±8.1%, respectively [17]. As shown in Fig 4B, only a small population of cells in adult MNCs was CD133-positive. The percentage of CD133^+^/DAPI^+^ cells in adult MNCs was 6.1±1.1% (n = 3), which was significantly lower than that in UCB-MNCs or SCE-UCBCs (adult MNCs, 6.1±1.1% (n = 3) vs. UCB-MNCs, 54.7±10.8% (n = 4), p<0.05; adult MNCs, 6.1±1.1% (n = 3) vs. SCE-UCBCs, 77.0±8.1% (n = 4), p<0.01, Table 4). These results indicate that adult MNCs contain a much smaller population of stem cells compared to UCB-MNCs or SCE-UCBCs. Since the highly sulfated E-unit (ΔDi-di4, 6S) was only detected in SCE-UCBCs (Table 2), there is a possibility that the E-unit might have some biological function in UCBCs.

**Table 4:** The number of cells examined.

	Total cell number (1x10^6^ cells)	CFU-GM assay (%)	Number of expected CF cells (1x10^4^ cells)	CD133^+^/DAPI (%)	Number of expected CD133^+^ cells (1x10^6^ cells)
SCE-UCBC^a^	14.7±7.5	20.2±1.04^d^	296	77.0±8.1^d^	11.3
UCB-MNC^b^	3.8	0.9±0.12^d^	3.42	54.7±10.8 ^d^	2.08
Adult MNC^c^	10.3±2.5	0.035±0.0075	0.36	6.1±1.1	0.63

^a^stem-cell-enriched umbilical cord blood cells (n = 3),

^b^mononuclear cells derived from rat umbilical cord blood (collected from 70 pups),

^c^mononuclear cells derived from peripheral blood of adult rat (n = 3).

^d^cited from Nakanishi et al., 2017.

### Effect of CS/DS and/or CS-E in the colony-forming cell assay

**Fig 5:**
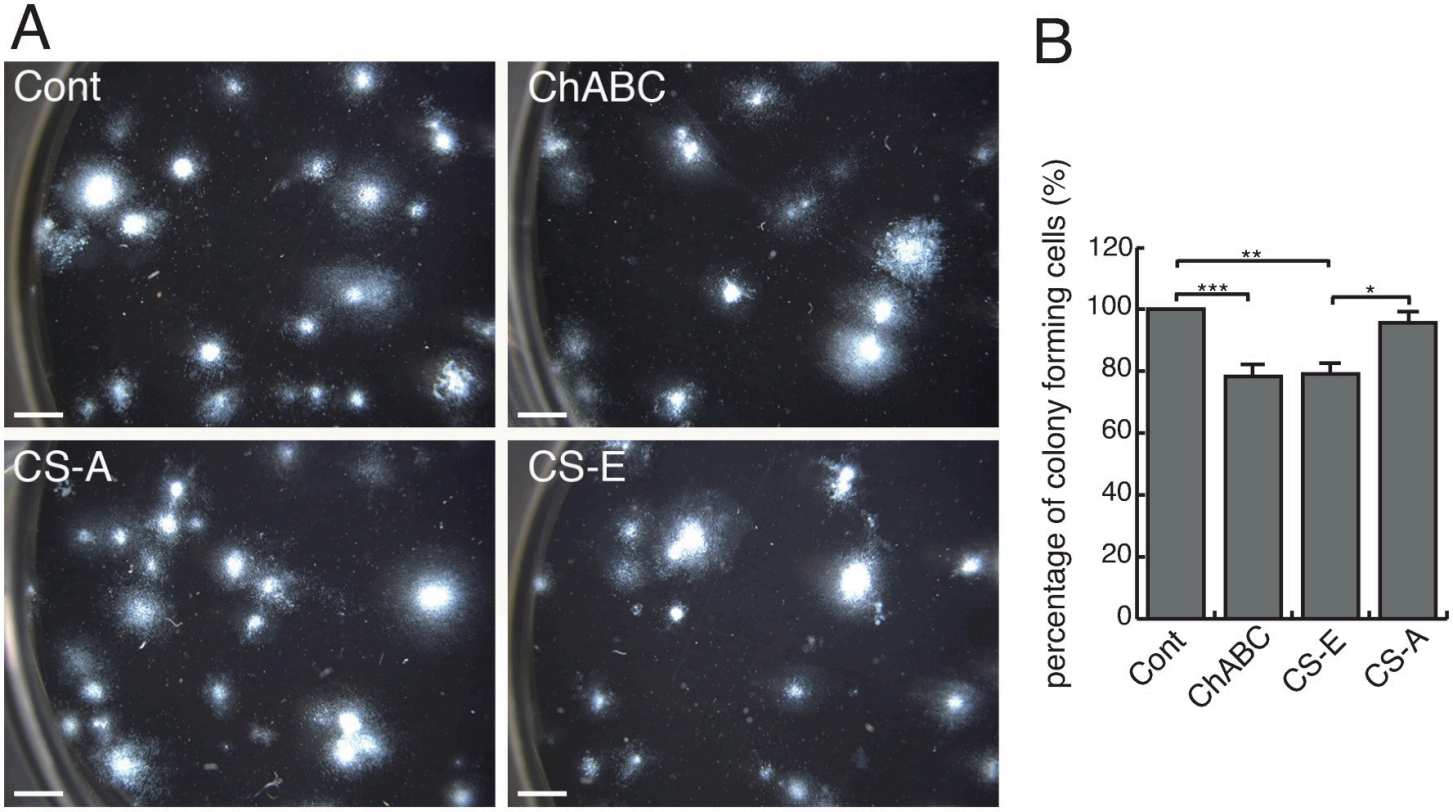
Effect of CS/DS in the CFU-GM assay. (A) Representative photographs of the CFU-GM assay in the control (Cont), in culture with Chase ABC (ChABC), with CS-A, or with CS-E. Bar, 2 mm. (B) The percentage of colony-forming cells. Data are expressed as means±SEM. *p<0.05, **p<0.01, ***p<0.001. (control, n = 4; ChABC, n = 4; CS-A, n = 3; CS-E, n = 4).

To examine the involvement of CS/DS and/or CS-E in biological activities of UCBCs, we carried out a CFU-GM assay with Chase ABC, CS-E, or CS-A. As shown in Fig 5A, various sizes and shapes of colonies were observed in each culture. The total number of colonies seemed to be slightly fewer in culture with ChABC and with CS-E compared to the control (PBS). A similar tendency was obtained in repeated experiments. The percentage of colony-forming cells in culture with Chase ABC treatment was less than that in the control (PBS, 100±0%, n = 4, vs. ChABC, 79.4±2.9%, n = 4, p<0.001, Fig 5B). In addition, the percentage of colony-forming cells in culture with CS-E was less than that in the control (PBS) or CS-A (CS-E, 81.1±3.2%, n = 4, vs. PBS, 100±0%, n = 4, p<0.01; CS-E, 81.1±3.2%, n = 4, vs. CS-A, 95.6±3.8%, n = 3, p<0.05, Fig 5B). This result suggests that CS/DS may have certain biological effects and that those of CS-E are distinct from those of CS-A in UCBCs.

## Discussion

Perinatal hypoxia–ischemia (HI) remains a tragic cause of neonatal death and/or severe neurological disorders [17, 26]. Since therapeutic hypothermia for treating HI brain injury is not sufficiently effective in severe cases [27, 28], a novel approach such as the use of stem cell therapy has been anticipated [12, 17]. We previously demonstrated that intracerebroventricular injection of neural stem/progenitor cells (NSPCs) together with chondroitinase (Chase) ABC significantly decreased the degree of cerebral infarction after perinatal HI injury in a rat model [29, 30]. However, intracerebroventicular injection of NSPCs presents technical challenges as well as ethical problems with regard to clinical application, and the understanding of the public with regard to the collection of NSPCs from human embryos would be difficult to achieve. Intracerebroventricular injection is an invasive injection technique which is largely used for biomedical research and can be rarely used for drug application in the treatment of human cerebral gliomas [31]. On the other hand, umbilical cord blood cells (UCBCs) as a possible source of stem cells, are hypo-immunogenic [32, 33] and can be administered intravenously. Clinical trials using autologous human UCBCs for neonatal hypoxic-ischemic encephalopathy (HIE) have begun [34, 35], and the feasibility of this approach has been reported [36, 37]. However, UCBCs have not been fully characterized. In the present study, we investigated the characteristics of CS/DS in stem-cell-rich populations derived from UCBCs, especially with regard to disaccharide unit composition. As far as we know, this is the first report to investigate the disaccharide composition of CS/DS or HS in MNCs or in a stem-cell-rich population derived from UCBCs.

First, we confirmed the existence of CS in vasculatures of umbilical cord and placenta by immunohistochemistry using a monoclonal antibody. CS was detected particularly in intima and media of umbilical arteries (Fig 1). We next examined the immunocytochemistry of SCE-UCBCs with unsulfated CS stub antibody (1B5, a stub antibody against the O-unit) which confirmed the existence of CS in some SCE-UCBCs (Fig 2). Although the percentage of 1B5-positive cells seemed to be low (6.5±1.9%), the major component of CS in SCE-UCBCs was the A-unit (ΔDi-4S, more than 80%) and the O-unit (ΔDi-0S) represented only 4–13% (Table 2). Therefore, the percentage of 1B5 (O-unit)-positive cells does not always represent the percentage of CS-harboring cells.

Next, we performed a disaccharide composition analysis, which revealed that the main CS/DS disaccharide unit in UCBCs and/or MNCs was the A-unit (Table 2). The HS content was smaller than the CS content in UCBCs and/or MNCs (Tables 2 and 3, S1 and S2 Tables in S1 File), which may be consistent with previous findings with human leukocytes which showed that CS is much more abundant than HS [38]. In addition, the CS content in lymphocytes was much less than that in myeloid cells [38]. Because freshly isolated MNCs usually contain many low-density lymphocytes [39], our observation that the CS content in MNCs was less than that in SCE-UCBCs may be owing to the difference in cell populations.

The amount of the A-unit on digestion with Chase ABC (which digests CS and DS) was larger than that on digestion with Chase ACII (Table 1), indicating that SCE-UCBCs presumably harbor CS/DS hybrid chains. CS/DS hybrid chains have often been found in various tissues such as embryonic pig brains and shark skins [6]. CS/DS hybrid chains in embryonic pig brain are reported to interact with pleiotrophin, a heparin-binding growth factor, and to have biological activities such as neurite-outgrowth-promoting activity in embryonic mouse hippocampal neurons [40]. CS/DS hybrid chains in UCBCs might also have biological activities involving interaction with growth factors.

Interestingly, the highly sulfated E-unit was only detected in SCE-UCBCs (Table 2). SCE-UCBCs are expected to include many colony-forming cells (about 3.0 x 10^6^ cells), while UCB-MNCs and adult MNCs would have 3.4 x 10^4^ and 3.6 x 10^3^ colony-forming cells, respectively (Table 4). In addition, CD133^+^ cells, another stem cell marker, are abundant in SCE-UCBCs (Table 4), suggesting that SCE-UCBCs contain a stem-cell-rich population. It is likely that the highly sulfated E-unit containing CS might be involved in some biological function on HSCs. CS-E is known to bind to heparin-binding growth factors and affect the biological activity of neural cells [7, 8]. CS polysaccharides derived from E14 rat telencephalon contain a small portion of DS and the E-unit and commercial preparations of DS (CS-B) and an E-type of highly sulfated CS promote fibroblast growth-factor-2-mediated proliferation of NSPCs [3]. CS-E in endothelial cells binds to vascular endothelial growth factor (VEGF)-A and has been shown to have a significant role in regulation of angiogenesis [41]. Indeed, the percentage of colony-forming cells, under the condition that the culture contained CS-E, was lower than that of the control or CS-A (Fig 5B). In our CS-E-containing culture, some growth factors may have been trapped by the CS-E added to the medium, which would have prevented colony formation.

It was previously postulated that CS may have an important role in hematopoiesis. Disruption of a gene encoding the rate-limiting CS-synthesizing enzyme *N*-acetylgalactosaminyltransferase-1 (T1) in mice causes about a 50% decrease in the amount of CS in bone marrow (BM), and T1-deficient mice have a higher number of CFU-GMs in BM cells compared to wild type mice [42]. The addition of DS (CS-B) together with thrombopoietin increased the number of CFU-Meg cells in a culture of CD34^+^ cells purified from human peripheral blood [43]. In the present study, treatment with Chase ABC reduced the percentage of colony-forming cells (Fig 5B), indicating that CS/DS might be involved in some biological activity such as the proliferation of HSCs. Although a colony-forming cell assay is useful to examine culture protocols for stem cell expansion, most progenitors detected in this type of assay are not considered to be HSCs with a long-term reproductive ability [44]. On the other hand, recent technological advances have revealed significant HSC heterogeneity and challenged the classical view of HSC biology [45]. Considering the heterogeneity of HSCs, it is possible that CS/DS and /or CS-E might have some specific role in certain kinds of hematopoietic lineages. CS/DS polysaccharide is also highly complex and heterogenous in its structure and sulfation pattern [4, 5]. More studies are needed to clarify the potential role of CS/DS on hematopoiesis in order to advance the field of stem cell glycobiology.

## Supporting information

S1 File Supporting information—Contains all the supporting tables and figures. (PDF)

## Abbreviations

BM: bone marrow
CFU-GM: colony-forming unit-granulocyte, macrophage
Chase: chondroitinase
CNS: central nervous system
CS: chondroitin sulfate
DS: dermatan sulfate
GAG: glycosaminoglycan
GalNAc: *N*-acetyl-D-galactosamine
GlcA: glucuronic acid
HA: hyaluronic acid
HI: hypoxic-ischemic
HIE: hypoxic-ischemic encephalopathy
HS: heparan sulfate
HSC: hematopoietic stem cell
IdoA: iduronic acid
MNC: mononuclear cell
MSC: mesenchymal stem cell
NSPC: neural stem/progenitor cell
PBS: phosphate-buffered saline
SCE-UCBC: stem-cell-enriched umbilical cord blood cell
UCBC: umbilical cord blood cell
