# Supplementary material for: Characterization of chondroitin sulfate in stem cells derived from umbilical cord blood in rats
Source: PLoS One. 2022 Jan 25;17(1):e0262854. doi: 10.1371/journal.pone.0262854 (PMC8789104; doi:10.1371/journal.pone.0262854)
Supplement: S1 File — (PDF) [file pone.0262854.s001.pdf]

Supporting Information

Characterization of chondroitin sulfate in stem cells derived from umbilical cord blood in rats.

by Keiko Nakanishi, Kyohei Higashi, Toshihiko Toida, and Masato Asai

Supplementary Information

Isolation and culture of rat mononuclear cells (MNCs) derived from UCBCs

MNCs were isolated using Ficoll-Conray solution (Lymphosepar I, Immuno-Biological Laboratories) or Ficoll-Paque PLUS solution (GE Healthcare) and were maintained in Stemline hematopoietic stem cell expansion medium (Stemline I, 0189, Sigma-Aldrich Co.) or StemSpan^{TM} serum-free expansion medium (#ST-09650, Stemcell Technologies) containing 8 mM glutamine (Gibco, Life Technologies), 50 U/mL penicillin G, 0.025 mg/mL streptomycin (Meiji Seika Ltd.), and 0.1 mM 2-mercaptoethanol, under 95% ambient air, 5% CO_2, and an H_2O-saturated atmosphere at 37 °C. The cells were expanded using a cocktail of growth factors containing 20 ng/mL of rat stem cell factor, rat thrombopoietin, rat IL-6, and human Flt-3 ligand (all purchased form PeproTech Inc.) and then cryopreserved [11].

Quantification of immunocytochemical staining

Quantification of immunocytochemical staining was performed using the public domain Image J program (Rasband, 1997; Schneider et al., 2012) after taking photomicrographs as described previously [17]. The photomicrographs in each experiment were taken under the same conditions and the images were changed into gray scale with Photoshop software (Adobe Systems Inc., San Jose, CA, USA), and analyzed at an equal threshold in Image J. Fluorescent images of at least 10 fields per coverslip (x40 objective) were obtained using a fluorescence microscope (Nikon, Eclipse E800), and the acquired images were subjected to particle analyses using Image J software. The positive cells in each field were counted and the number was expressed as the percentage of the total number of DAPI-positive cells. Three (for CD133 staining, adult MNCs) or four (for 1B5 staining, UCB-MNCs) independent experiments were carried out.

Quantitative analyses of GAGs

Microscale isolation of GAGs was performed according to the method of Zhang et al. [29]. Briefly, the filtered extracts were purified on a Vivapure Q mini H spin column (Sartorius AG) which was centrifuged then washed three times with 450 µL of 0.2 M NaCl. Crude GAGs were then eluted from the column by washing two times with 500 µL of 16% NaCl. The GAGs were precipitated from the supernatant by the addition of four volumes of cold methanol for 16 h at 4 °C and were recovered by centrifugation at 11,000 x g for 30 min. The GAG samples were

incubated in a reaction mixture (35 μL) containing 28.6 mM Tris-acetate (pH 8.0) and 50 mIU of Chase ABC (and Chase ACII) for 16 h at 37 °C. Depolymerized samples were boiled and evaporated, and the unsaturated disaccharides of CS/DS were collected on an Amicon Ultra centrifugal Filter 30 K device (EMD Millipore). The remaining HS samples in the filters of the spin column were transferred to new microtubes and incubated in 16 μL of reaction mixture (pH 7.0) containing 1 mIU heparinase I, 1 mIU heparinase II, 1 mIU heparinase III, 31.3 mM sodium acetate and 3.13 mM calcium acetate for 16 h at 37 °C. Unsaturated disaccharide analysis of CS/DS and HS was performed by reversed phase ion-pair chromatography with sensitive and specific post-column detection. A gradient was applied at a flow rate of 1.0 mL/min on Senshu Pak Docosil (4.6 × 150 mm; Senshu Scientific, Tokyo, Japan) at 60℃. The eluent buffers were as follows: A, 10 mM tetra-*n*-butylammonium hydrogen sulfate in 12% methanol; B, 0.2 M NaCl in buffer A. Aqueous (0.5% (w/v)) 2-cyanoacetamide solution and 1 M NaOH were added to the eluent at the same flow rates (0.5 mL/min) by using a double plunger pump. The effluent was then monitored fluorometrically (excitation, 346 nm; emission, 410 nm).

References

1) Rasband, W.S. *ImageJ, U. S. National Institutes of Health, Bethesda, Maryland, USA* (1997-2012). Available at: http://imagej.nih.gov/ij/
2) Schneider, C.A., Rasband, W.S. & Eliceiri, K.W. NIH Image to ImageJ: 25 years of image analysis. *Nature Methods* **9**, 671-675 (2012).

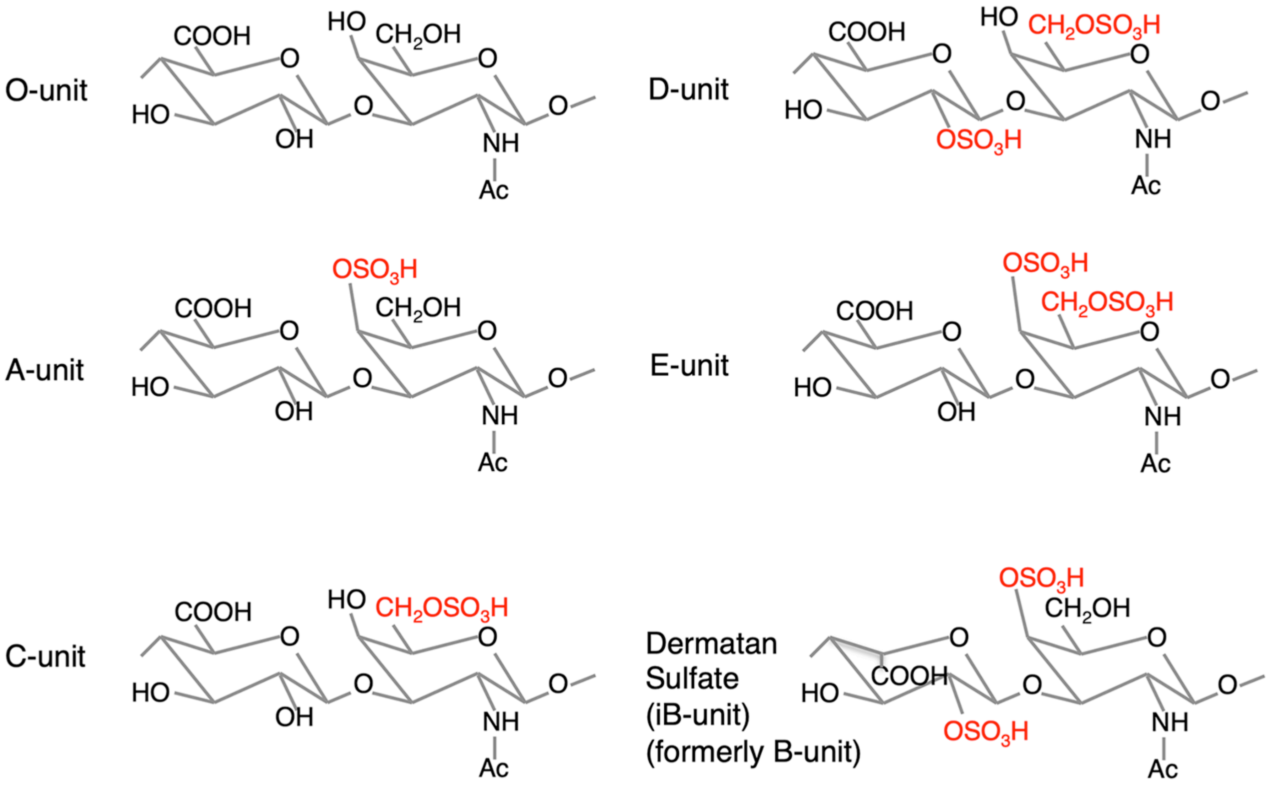

S1 Fig

Structure of disaccharide unit of chondroitin sulfate (CS)/dermatan sulfate (DS) chains.

The CS chain consists of repeating disaccharide units of glucuronic acid (GlcA) and *N*-acetylgalactosamine (GalNAc) with different numbers and positions of sulfation; namely, O, A, C, D, and E. Dermatan sulfate (DS, formerly CS-B), another isomeric variant of CS, has iduronic acid (IdoA)-containing disaccharide units instead of GlcA (iB-unit). The figure was made according to Lamari and Karamanos [5].

S1 Table Results of Disaccharide analyses (CS/DS)

CS/DS	Cell number ($1x10^6$ cells)	Unsaturated disaccharide units								ng/mg protein	Amount of protein (mg)	Amount of CS/DS (ng)
		ΔDi-0S (%)	ΔDi-4S (%)	ΔDi-6S (%)	ΔDi-UA2S (%)	ΔDi-di4,6S (%)	ΔDi-di2,4S (%)	ΔDi-di2,6S (%)	ΔDi-triS (%)			
UCBC 1	16	21.9	75.7	0.5	ND	1.9	ND	ND	ND	330.4	1009.9	333.7
UCBC 2	15	12.7	84.3	0.3	ND	2.7	ND	ND	ND	408.2	1147.3	468.3
UCBC 3	7	14.4	82	0.5	ND	3.1	ND	ND	ND	716.2	625.3	447.8
UCBC 4	22	12.4	83	0.3	ND	4.2	ND	ND	ND	508.7	1867.0	949.8
UCB-MNC[a]	3.8	13.7	86.3	0.0	ND	ND	ND	ND	ND	237.9	90.1	21.4
MNC1	13	ND	80.8	19.2	ND	ND	ND	ND	ND	76.6	142.3	10.9
MNC2	10	6.8	89.3	3.9	ND	ND	ND	ND	ND	149	142.3	21.2
MNC3	8	6.3	89.9	3.8	ND	ND	ND	ND	ND	235.8	79.1	18.7

[a]UCB-MNC(collected from 70 pups), ΔDi-0S, deoxy-α-L-*threo*-hex-4-enopyranosyluronic acid(ΔUA)(1→3)*N*-acetylgalactosamine (GalNAc); ΔDi-4S, ΔUA(1→3)GalNAc4S, where S is sulfo; ΔDi-6S, ΔUA (1→3) GalNAc6S; ΔDi-UA2S, ΔUA2S (1→3) GalNAc; ΔDi-di4,6S, ΔUA (1→3) GalNAc4S6S; ΔDi-di2,4S, ΔUA2S (1→3) GalNAc4S; ΔDi-di2,6S, ΔUA2S (1→3) GalNAc6S; ΔDi-TriS, ΔUA2S (1→3) GalNAc4S6S; ND, not detected.

S2 Table Results of Disaccharide analyses (HS)

HS	Cell number ($1x10^6$ cells)	Unsaturated disaccharide units						ng/mg protein	Amount of protein (mg)	Amount of HS (ng)
		ΔDi-0S (%)	ΔDi-NS (%)	ΔDi-6S (%)	ΔDi-NS6S (%)	ΔDi-NSUA 2S (%)	ΔDi-triS (%)			
UCBC1	16	20.6	34.2	6.4	3.9	11.9	23.0	150.7	1009.9	152.2
UCBC2	15	19.2	27.3	7.0	4.9	17.4	24.1	108.3	1147.3	124.2
UCBC3	7	25.9	43.2	5.8	2.5	8.4	14.2	177.7	625.3	111.1
UCBC4	22	18.5	35.5	5.0	2.9	14.1	24.0	154.0	1867.0	287.4
UCB-MNC[a]	3.8	ND	ND	ND	ND	ND	ND	ND	90.1	ND
MNC1	13	19.0	81.0	ND	ND	ND	ND	5.6	142.3	0.8
MNC2	10	42.7	57.3	ND	ND	ND	ND	9.4	142.3	1.3
MNC3	8	100.0	ND	ND	ND	ND	ND	7.5	79.1	0.6

[a]UCB-MNC(collected from 70 pups), ΔDi-0S, ΔUA (1→4) *N*-acetylglucosamine (GlcNAc); ΔDi-NS, ΔUA (1→4) *N*-sulfated glucosamine (GlcNS); ΔDi-6S, ΔUA (1→4) GlcNAc6S; ΔDi-NS6S, ΔUA (1→4) GlcNS6S; ΔDi-NSUA2S, ΔUA2S (1→4) GlcNS; ΔDi-TriS,ΔUA2S (1→4) GlcNS6S; ND, not detected.

Supporting Information

The values behind the means, standard deviations and other measures reported;

Fig. 2 (text, line 235); raw data of percentage of 1B5-positive cells

Fig.2 raw data of 1B5 staining														
y25				609Di0S				630Di0S				721Di0S		
file name	dapi	1B5		file name	Dapi	1B5		file nam	Dapi	1B5		file name	Dapi	1B5
1d	197	4		1d	40	3		34d	34	1		70d	40	1
3d	204	1		4d	25	1		37d	31	4		73d	36	3
5d	175	3		7d	33	1		40d	41	2		76d	44	1
9d	221	5		10d	37	3		43d	44	4		79d	13	1
11d	241	7		13d	33	2		46d	35	3		82d	23	2
13d	278	4		16d	25	3		49d	28	2		85d	16	0
15d	248	10		19d	31	5		52d	19	7		88d	44	2
17d	217	4		22d	26	1		55d	20	5		91d	78	3
19d	220	5		25d	26	2		58d	27	3		94d	34	2
21d	198	4		28d	33	5		61d	40	4		97d	15	2
23d	237	4		31d	29	3		64d	34	3		100d	42	2
								67d	49	4				
sum	2436	51		sum	338	29		sum	402	42		sum	385	19
1B5/dapi(%)		2.094				8.58				10.45				4.935

Fig.2 1B5 summary		
	1B5/DAPI(%)	
y25	2.094	
609Di0S	8.58	
630Di0S	10.45	
721Di0S	4.935	
average	6.514	
sd	3.732	
sem	1.866	

Table 1, 2, and 3; Raw data were shown in S1 and S2 Table.

Table 4; SCE-UCBC & UCB-MNC; These were cited from Nakanishi et al, 2017 [ref. 17].

Table 4 ; Raw data of CFU-GM assay in adult MNC

Table 4/ Fig.4	adultMNC-CFU-GMassay raw data				
seeded cell #	2x10e4 cell/well				total cell #
	colony #	average	(%)		x10e6
MNC3-1	11	8.5	0.0425		13
MNC3-2	6				
MNC4-1	2	4	0.02		10
MNC4-2	6				
MNC5-1	10	8.5	0.0425		8
MNC5-2	7				
		mean	0.035	mean	10.33333
		SD	0.01299	SD	2.516611
		SEM	0.0075	SEM	1.452966

Adult MNC; raw data of CD133 staining

Table4-adultMNC-CD133 raw data										
624-M				922-M1				922-M2		
file name	DAPI	CD133		file name	DAPI	CD133		file name	DAPI	cd133
105d	57	5		1d	63	2		21d	167	3
107d	46	7		3d	86	8		23d	177	8
109d	87	4		5d	98	7		25d	174	11
111d	65	10		7d	86	5		27d	165	5
113d	103	14		9d	84	4		29d	157	7
115d	87	4		11d	74	5		31d	168	5
117d	101	8		13d	92	5		33d	177	9
119d	122	7		15d	70	2		35d	136	7
121d	104	7		17d	88	3		37d	165	11
123d	65	3		19d	78	4		39d	177	8
sum	837	69			819	45			1663	74
CD133/DAPI(%)		8.244				5.495				4.45

Table4 CD133/DAPI(%)		
624-M	8.244	
922-M1	5.495	
922-M2	4.45	
average	6.063	
sd	1.96	
sem	1.131	

- The values used to build graphs;

Fig. 5; Raw data of CFU-GM assay

Fig.5 CFU-GM assay Raw data										
609CFU	1x10e4 cell/well s		CFU-GM assay			630CFU	1x10e4cells/well seed			
well	colony #	average	(%)			well	colony #	average	%	
pbs1	63	62	0.62	100		630pbs1	86	85.5	0.855	100
pbs2	61					630pbs2	85			
ChABC1	54	51.5	0.515	83.06		630chabc1	78	73	0.73	85.38
ChABC2	49					630chabc2	68			
CSE1	59	54	0.54	87.1		630cse1	75	68.5	0.685	80.12
CSE2	49					630cse2	62			
						630csa1	80	88	0.88	102.9
						630csa2	96			
707CFU	5x10e3 cell/well seed					721CFU	1x10e4 cell/well seed			
well	colony #	average	%			well	colony #	average	%	
707pbs1	37	36.5	0.73	100		721pbs1	62	67.5	0.675	100
707pbs2	36					721pbs2	73			
707chabc1	25	28	0.56	76.71		721chabc1	53	49	0.49	72.59
707chabc2	31					721chabc2	45			
707cse1	27	26.5	0.53	72.6		721cse1	55	57	0.57	84.44
707cse2	26					721cse2	59			
707csa1	35	33	0.66	90.41		721csa1	63	63	0.63	93.33
707csa2	31					721csa2	63			

Fig.5 CFU-GM assay summary				
	PBS	ChABC	CSE	CSA
609	100	83.06	87.1	
630	100	85.38	80.12	102.9
707	100	76.71	72.6	90.41
721	100	72.59	84.44	93.33
	PBS	ChABC	CSE	CSA
mean	100	79.44	81.07	95.56
sem	0	2.926	3.166	3.779
N	4	4	4	3
